# Associations of greenspace use and proximity with self-reported physical and mental health outcomes during the COVID-19 pandemic

**DOI:** 10.1371/journal.pone.0280837

**Published:** 2023-03-01

**Authors:** Janelle R. Edwards, Jeromy W. Gotschall, Jane E. Clougherty, Leah H. Schinasi

**Affiliations:** 1 Department of Environmental and Occupational Health, Dornsife School of Public Health, Drexel University, Philadelphia, Pennsylvania, United States of America; 2 Urban Health Collaborative, Dornsife School of Public Health, Drexel University, Philadelphia, Pennsylvania, United States of America; 3 Perelman School of Medicine, University of Pennsylvania, Philadelphia, Pennsylvania, United States of America; Universidad Nacional Autonoma de Nicaragua Leon, NICARAGUA

## Abstract

Research has shown that the COVID-19 pandemic affected individual’s mental and physical health. The aim of this study was to estimate associations between greenspace use and proximity with perceived mental and physical health during the COVID-19 pandemic. We surveyed metropolitan Philadelphia residents, October 20–December 1, 2020, about walking time to the nearest greenspace from their home, frequency of greenspace use in the past 30 days, change in frequency of greenspace use during the COVID-19 pandemic, and perceived physical and mental health outcomes. We ran unadjusted and adjusted log-binomial regression models to derive Risk Ratio (RR) and 95% Confidence Intervals (CI) estimates of associations of loneliness, physical and mental health outcomes with: (1) self-reported walking time to nearest greenspace; (2) reported greenspace use frequency; and (3) changes in greenspace use frequency. Of 485 survey participants, 244 (51.4%) reported feeling lonelier, 147 (31.37%) reported higher perceived stress, 261 (54.9%) reported worsened mental health, and 137 (28.7%) reported worsened physical health during vs. before the start of pandemic-restrictions in mid-March of 2020. After adjustment for gender, age, and change in financial status, RR estimates suggested modest protective associations between visiting greenspaces more frequently during vs. before the pandemic and worsened mental (RR: 0.84, 95% CI: 0.70–1.00), and physical health (RR 0.77, 95% CI: 0.56–1.10), and loneliness (RR: 0.91, 95% CI: 0.75–1.1) and perceived stress (RR 0.83, 95% CI: 0.61–1.13). Shorter walking distances to the nearest greenspace were associated with reduced risk of reporting worsened physical health and higher perceived stress; however, living shorter walking distances from greenspace were not associated with protection against worsened mental health or loneliness. These results suggest that active greenspace use may provide mental and physical health protection, particularly during a stressful public health crisis.

## Introduction

A growing body of empirical evidence suggests that greenspace, defined as land covered with natural vegetation such as grass, shrubs, and trees, or built environments such as parks and green recreational facilities [1], may provide important mental and physical health benefits [1–12]. Research suggests that greenspace is associated with higher levels of social cohesion and physical activity [13–21], and better self-rated health [2]. In addition, greenspace may have psychological restorative properties. Stress reduction theory posits that environmental experiences, such as viewing nature or vegetation, may prevent stress responses by evoking positive emotions and blocking negative ones [13, 22]. The potential psychosocial restorative benefits of greenspace have been supported by previous experimental [23] and observational [12] studies.

Recently, the novel coronavirus (SARS-CoV-2) represented one of the greatest physical and mental health challenges that our generation had ever before seen or experienced. To prevent viral transmission, public health agencies enacted a variety of social distancing measures [24]. This public health crisis and the associated social distancing measures had tremendous adverse implications for physical and mental well-being, including increased feelings of loneliness, anxiety, and decreases in physical activity [25, 26].

We leveraged the COVID-19 pandemic to empirically explore the hypothesis that greenspace use or exposure provides physical and psychological health benefits. We hypothesized that greater frequency of greenspace utilization, and/or greater proximity to greenspace, would reduce the adverse mental and physical health consequences of the COVID-19 pandemic. To explore our hypotheses, we surveyed adult metropolitan-Philadelphia area residents about their greenspace use and physical and mental-health during the COVID-19 pandemic. We chose the Philadelphia region as our study location because it is one of the largest major cities in the United States [27] that has varying densities of greenspace [28]. The mayor of Philadelphia, Jim Kenney, mandated that all non-essential businesses shut down starting March 16^th^, 2020 [29]. While playgrounds were also mandated to close at that time, green spaces, such as, Wissahickon Valley Park, an 1,800 acre gorge with dozens of miles worth of trail [30], or Bartram’s Garden, a 50-acre public garden, remained open [30]. Thus, residents of Philadelphia and nearby areas had the opportunity to visit parks and gardens for outdoor refuge and distraction during this challenging time.

## Materials and methods

### Study setting

Our study setting was the metropolitan Philadelphia region, which we defined as any zip code within the combined statistical area (CSA) of Philadelphia, Camden and Vineland counties. CSAs are geographic delineations made by the US Census Bureau. They contain a core urbanized cluster in addition to adjacent counties that have a high degree of social and economic exchange [31].

### Ethical statement and recruitment

This research involves human participants and was approved by Drexel University’s Office of Human Research Protection (IRB protocol # 2008008052). Before they were able to begin responding to the survey questions, participants read a brief introductory page, which served as the mechanism for receiving participants’ informed consent. Specifically, the introductory page provided details about the study, eligibility criteria, and contact information for the Principal Investigator and the Institutional Review Board. Participants were also told that their participation in the survey was completely voluntary, that their responses would be anonymous, that they were not obligated to respond to the questions or participate, and that there were no risks or benefits associated with completing the survey. Participants were informed that, by proceeding to the next page of the survey, they were indicating their informed consent. The full text of the information that all eligible participants read before indicating their informed consent is given in the S1 File.

In Fall of 2020, we recruited adults ages 18 years or older who lived in the metropolitan Philadelphia region, spoke English, and had access to an internet connection and device (e.g., smart phone, computer, tablet) with which to complete a structured, web-based survey. The survey was advertised through social networks, through emails to community listservs, and by word of mouth. The survey opened on October 20^th^, 2020 and closed on December 1^st^, 2020. A copy of the full survey is presented in the S2 File.

### Questionnaire

The survey contained structured questions on sociodemographics (age, gender identity, race, Hispanic/Latinx ethnicity, household income, education level), residential zip code, greenspace use frequency, perceived walking distance to nearest greenspace, and reasons for not using greenspaces. Participants were asked to report on changes in feelings of loneliness, mental health, physical health, and financial status, comparing the current time to before the start of the COVID-19 restrictions in mid-March of 2020. We also asked respondents to report their stress, experienced in the past 30 days, using a modified version of the validated Perceived Stress Scale [32]. The scale had four items, 2 of which were positive statements. Respondents were asked to rate their ability to cope with stressors and the frequency of negative feelings about life events in the past 30 days. Specifically, participants were asked “How often have you felt that you were unable to control the important things in your life?”, “How often have you felt confident about your ability to handle your personal problems?”, “How often have you felt that things were going your way?”, and “How often have you felt difficulties were piling up so high that you could not overcome them?”. Each item was rated using a 5-point Likert scale (0 = never, 1 = almost never, 2 = sometimes, 3 = fairly often, 4 = very often); the score for the 2 positive statements were reversed and added to yield a maximum score of 16. A higher score indicated more perceived stress.

Specific questions on greenspace use or proximity were the following: (1) frequency of visits to greenspace in the past 30 days (henceforth, denoted greenspace use [visits/week]); (2) change in greenspace use, comparing the current time period to mid-March before the start of the COVID-19 restrictions (henceforth, denoted change in greenspace use); and (3) perceived walking time (in minutes) to the nearest greenspace from residence (henceforth, denoted perceived greenspace proximity). Because we were primarily interested in capturing changes in well-being that occurred after the COVID-19 pandemic restrictions, nearly all questions asked participants to compare their current situation to the period before mid-March 2020. We did not provide a specific March date, because although city-wide restrictions started on March 16, some survey participants may have perceived the start of the restrictions slightly earlier or later than this date, depending upon their personal work or living situation. In addition, to reduce concerns about seasonal confounding, we asked participants to report on changes in greenspace use compared to the same time in the previous year. Survey questions, response options, and our terminology are summarized in Table 1.

**Table 1:** Key exposure and outcome variables.

Survey question	Response Options	Manuscript terminology
**How long does it take you to walk to the green or natural space that is closest to your home?**	Fewer Than Five Minutes 5–9 Minutes 10–19 Minutes 20–30 Minutes More than 30 Minutes No Green/ Too Far to Walk	Perceived greenspace proximity
**Compared to before the start of the Coronavirus outbreak in mid-March, would you say that you visit green or natural spaces…** **Compared to this time last year (12 months ago), would you say that you visit green or natural spaces…**	Less often The same amount More often	Change in greenspace use
**Over the past 30 days, how often have you visited a green or natural space?**	More than 4 times per week 2–4 times per week 1–2 times per week 1–2 times per month Almost never	Greenspace use

### Statistical analysis

We used log-binomial regression models to derive Risk Ratio (RR) and 95% Confidence Interval (CI) estimates of association of perceived stress and changes in loneliness, mental health, and physical health with: (1) perceived greenspace proximity, (2) greenspace use, and (3) change in greenspace use. We specified models using a log-binomial distribution because the outcome variables in this analysis were common, making the use of logistic regression models inappropriate [33]. Variables representing changes in mental and physical health were coded as binary (0: stayed the same or improved [REF], 1: became worse). Perceived stress was also coded as binary (0: scale measures 0–7 [REF], 1: scale measures 8–16). We categorized change in greenspace use as a three-level categorical variable (0: less often [REF], 1: the same amount, 2: more often). We coded perceived greenspace proximity as a binary variable (0: more than 10 minutes [REF], 1: less than 10 minutes). Lastly, we coded greenspace use as a three-level categorical variable, where 0: less than 2 times a month [REF], 1: 1–4 times a week, and 2: more than 4 times week.

In models, perceived stress, and change in mental health, loneliness, or physical health served as the dependent variables (each modeled separately). The independent variables, examined separately, were greenspace use, change in greenspace use, or perceived greenspace proximity. Model set 1 derived unadjusted estimates of association. Model set 2 included terms for categorical age (18–24, 25–34, 35–44, 45–54, 55–64, 65+), financial status change (gotten worse vs. stayed the same/ improved during pandemic), and gender identity (non-binary, male, female). In set 3, we adjusted for perceived greenspace proximity in models with either greenspace use or change in use as the independent variable and adjusted for greenspace use in models with greenspace proximity as the independent variable. The rationale for including mutual adjustment for the other greenspace measure was to derive estimates of the independent effects of either perceived proximity or greenspace use. We did not adjust for race, ethnicity, education level, or household income, which did not vary markedly across survey participants. All covariates were selected based on *a priori* hypotheses that they might confound associations between the greenspace metrics and outcomes. We used a Pearson Correlation matrix to measure the correlation and direction of association between health outcomes.

As a sensitivity analysis, we reran the change in greenspace use models using the variable that compared use to one year ago, rather than to mid-March 2020. As an additional sensitivity analysis, we reran all models (crude and adjusted) to account for urbanicity by incorporating a Philadelphia status covariate. Philadelphia status was coded as a binary term (1: resident zip codes that fell within Philadelphia, 2: resident zip codes that fell outside of Philadelphia).

We report estimates as Risk Ratios and 95% CIs; we interpret results as significant based on the magnitude and direction of the estimate, and the width of the confidence limit. All analysis was conducted using SAS version 9.4.

## Results

### Study participants

Between October 20, 2020 and December 1, 2020, 488 metropolitan Philadelphia area residents participated in the survey. After excluding three participants who were under age 18 and thus ineligible, the final analytic sample included 485 people. Table 2 presents descriptive statistics on survey participants. Survey participants were majority white (88.5%), women (70.2%) and had at least Bachelor’s degree (91.8%). Approximately half of participants reported having an annual household income of $100,000 or greater, and reported no financial impacts during the pandemic. The majority of participants reported that their mental health and loneliness became worse during the pandemic (54.9% and 51.4% respectively); however, only a third reported that their physical health became worse or had higher perceived stress (28.7% and 31.3% respectively). In addition, out of the 485 participants, 20.2% reported experiencing COVID-19 symptoms; however, only a small percentage (2.8%) reported that they had ever tested positive for COVID-19.

**Table 2:** Sociodemographic characteristics and health outcomes among survey participants.

		N (%)
**Age (years)**		
	18–34	159 (33.9)
	35–54	147 (31.4)
	55+	163 (35.8)
**Gender identity**		
	Man	132 (28.6)
	Woman	325 (70.2)
	Non-binary	5 (1.1)
**Racial identity**		
	White	371 (88.5)
	Black	40 (9.6)
	Other	8 (1.9)
**Educational Attainment**		
	Below a Bachelor’s Degree	38 (8.2)
	Bachelor’s Degree or higher	428 (91.8)
**Household Income (US Dollars)**		
	0–49,000	100 (22.8)
	50,00–99,999	104 (23.7)
	100,00–169,99	125 (28.5)
	≤ 170,000	110 (25.0)
**COVID-19 Outcomes**		
	Experiencing Symptoms	98 (20.2)
	Tested Positive	14 (2.8)
**Financial Change** ^1^		
	Gotten Worse	81 (17.0)
	No Different	309 (64.9)
	Improved	86 (18.1)
**Mental Health Change** ^1^		
	Gotten Worse	261 (54.9)
	No Different	182 (38.3)
	Improved	32 (6.7)
**Physical Health Change** ^1^		
	Gotten Worse	137 (28.7)
	No Different	254 (53.3)
	Improved	86 (18.0)
**Loneliness Change** ^1^		
	Gotten Worse	244 (51.4)
	No Different	12 (2.5)
	Improved	219 (46.1)
**Perceived Stress Scale** ^2^		
	0–7	323 (68.7)
	8–16	147 (31.3)

^1^Change variables represent the participant’s perceived change at the time of survey response, relative to before the middle of March in year 2020.

^2^Perceieved stress was measured using a using a modified version of the validated Perceived Stress Scale. The scale had four items. For more detail refer to the questionnaire section.

### Health measures

Pearson correlation showing bivariate relationships between the mental and physical health outcomes are given in S1 Table, respectively. Overall, the physical and mental health outcome variables were only moderately, positively correlated. The highest correlations were between worsened loneliness and worsened mental health (r^2^: 0.50), and between higher perceived stress and worsened mental health (r^2^: 0.36). Worsened physical health was moderately, positively correlated with worsened mental health (r^2^: 0.27) and higher perceived stress (r^2^: 0.22), and modestly correlated with worsened loneliness (r^2^: 0.14).

### Associations between greenspace measures and changes in mental health

Table 3 shows RR (95% CI) estimates of association between the greenspace measures and worse mental health during the pandemic vs. before. In unadjusted models, compared to those who reported using greenspaces less frequently during the pandemic, those who reported using greenspaces with the same frequency had 33% lower risk of reporting worse mental health (95% CI: 0.53–0.85) and those who reported greater frequency of use during the pandemic had 22% lower risk (95% CI: 0.66–0.93). Additional adjustment for gender, age, financial status change, as well as perceived greenspace proximity, estimates were further attenuated to the null but remained protective (RR, 95% CI for same use compared to pre-pandemic: 0.74, 95% CI: 0.58–0.95; RR, 95% CI for more greenspace use: 0.83, 95% CI: 0.70–1.0). Associations between greenspace proximity and mental health change were close to null. Estimates of association between greenspace use in the past 30 days and change in mental health suggested that higher frequency of greenspace utilization was associated with higher risk of worsened mental health during the pandemic; however, estimates were imprecise and crossed the null.

**Table 3:** Estimates of association between the greenspace measures and worsened mental health, comparing the survey period to before the start of pandemic-related social distancing measures in March of 2020^1^.

	Model Set 1		Model Set 2		Model Set 3	
	RR	95% CI	RR	95% CI	RR	95% CI
**Perceived proximity (Walking distance to nearest greenspace)**						
More than 10 minutes	1.00		1.00		1.00	
Less than 10 minutes	0.97	0.83–1.14	1.03	0.87–1.22	1.01	0.85–1.19
**Greenspace use (past 30 days)**						
Less than 2 times a month	1.00		1.00		1.00	
1–4 times a week	1.30	0.99–1.70	1.36	1.02–1.80	1.37	1.03–1.84
More than 4 times a week	1.19	0.89–1.60	1.27	0.93–1.72	1.28	0.94–1.75
**Greenspace use change** ^2^						
Less frequently	1.00		1.00		1.00	
No change in frequency	**0.67**	**0.53–0.85**	**0.74**	**0.58–0.95**	**0.74**	**0.58–0.95**
More frequently	**0.78**	**0.66–0.93**	0.84	0.70–1.00	0.83	0.70–1.00

^1^Model set 1 is unadjusted. Model set 2 is adjusted for age, gender identity, and financial status change. Model set 3 is adjusted for age, gender identity, and financial status change, and other greenspace metrics: Models for greenspace space use and change in use are adjusted for proximity, and models for perceived greenspace proximity is adjusted for greenspace use.

^2^Represents the participant’s perceived change at the time of survey response, relative to before the middle of March in year 2020.

Bolded RR and 95% CIs represent statistically significance (p-value <0.05).

### Associations between greenspace measures and changes in loneliness

Table 4 shows RR (95% CI) estimates of association between the greenspace metrics and worsened feelings of loneliness. In both unadjusted and adjusted models, compared to those who reported using greenspaces less frequently compared to before mid-March, those who reported using greenspaces with the same or greater frequency had lower risk of reporting decrements in loneliness, even after adjustment for gender, age, and financial status change (RR comparing those who reporting same use with those who decreased their use: 0.72, 95% CI: 0.55–0.95). Similar to mental health results, individuals who maintained their greenspace use patterns had greater protection against decrements in loneliness than those who increased greenspace use. A small proportion of individuals who reported living less than ten minutes walking distance to the nearest greenspace reported increased feelings of loneliness compared to those who lived more than ten minutes from the nearest greenspace (RR: 0.87, 95% CI: 0.73, 1.04 after adjustment for age, gender identity, financial status change, and greenspace use in the past 30 days), although the CIs crossed the null. Likewise, associations for greenspace frequency in the past 30 days were close to null and/or had very imprecise with wide CIs.

**Table 4:** Estimates of association between the greenspace measures and worsened feelings of loneliness, comparing the survey period to before the start of pandemic-related social distancing measures in March of 2020^1^.

	Model Set 1		Model Set 2		Model Set 3	
	RR	95% CI	RR	95% CI	RR	95% CI
**Perceived proximity (Walking distance to nearest greenspace)**						
More than 10 minutes	1.00		1.00		1.00	
Less than 10 minutes	**0.83**	**0.69–0.99**	0.88	0.74–1.05	0.87	0.73–1.04
**Greenspace use (past 30 days)**						
Less than 2 times a month	1.00		1.00		1.00	
1–4 times a week	1.07	0.82–1.38	1.04	0.81–1.33	1.08	0.84–1.39
More than 4 times a week	1.03	0.77–1.36	1.01	0.77–1.33	1.05	0.80–1.40
**Greenspace use change** ^2^						
Less frequently	1.00		1.00		1.00	
No change in frequency	**0.66**	**0.51–0.87**	**0.72**	**0.55–0.95**	**0.70**	**0.54–0.92**
More frequently	0.86	0.71–1.00	0.91	0.75–1.10	0.92	0.76–1.10

^1^Model set 1 is unadjusted. Model set 2 is adjusted for age, gender identity, and financial status change. Model set 3 is adjusted for age, gender identity, and financial status change, and other greenspace metrics: Models for greenspace space use and change in use are adjusted for proximity, and models for perceived greenspace proximity is adjusted for greenspace use.

^2^Represents the participant’s perceived change at the time of survey response, relative to before the middle of March in year 2020.

Bolded RR and 95% CIs represent statistically significance (p-value <0.05).

### Associations between greenspace measures and perceived stress

Table 5 shows RR (95% CI) estimates of associations between the greenspace metrics and higher levels of perceived stress, as measured by the modified stress scale. All greenspace measures RR estimates were suggestive of protective associations. In unadjusted models, compared to those who reported using greenspaces less frequently during the pandemic, those who reported using greenspaces with the same frequency had 31% lower risk of reporting higher perceived stress (95% CI: 0.47–1.01), and those who reported greater frequency of use during the pandemic had 28% lower risk (95% CI: 0.53–0.98). For perceived proximity, in unadjusted models, those who lived less than 10 minutes away from greenspace had a 24% less risk of perceived stress than those who lived more than 10 minutes walking distance from greenspace. Additional adjustment for gender identity, age, financial status change, and other greenspace metrics caused estimates of association to remain in the protective direction but to move closer to the null; CIs also crossed the null after additional adjustment. We also observed evidence of protective associations between frequency of greenspace use in the past 30 years and lower risk of perceived stress, although RR estimates were fairly imprecise with wide CIs. For example, after adjustment for age, gender identity, change in financial status, and greenspace proximity, compared to those who used greenspace least frequency (i.e., fewer than two times per month in the past 30 days), those who visited greenspace with moderate frequency (i.e., one to four times per week in the past 30 days), and with high frequency (i.e., more than four times per week) had 12% (95% CI: 0.63–1.23) and 26% (95% CI: 0.50–1.12) lower risk of perceived stress.

**Table 5:** Estimates of association between the greenspace measures and higher levels of perceived stress^1^^,^^2^.

	Model Set 1		Model Set 2		Model Set 3	
	RR	95% CI	RR	95% CI	RR	95% CI
**Perceived proximity (Walking distance to nearest greenspace)**						
More than 10 minutes	1.00		1.00		1.00	
Less than 10 minutes	**0.76**	**0.59–0.99**	0.81	0.62–1.06	0.82	0.63–1.07
**Greenspace use (past 30 days)**						
Less than 2 times a month	1.00		1.00		1.00	
1–4 times a week	0.88	0.62–1.24	0.90	0.64–1.27	0.88	0.63–1.23
More than 4 times a week	0.68	0.46–1.02	0.75	0.49–1.13	0.74	0.50–1.12
**Greenspace use change** ^3^						
Less frequently	1.00		1.00		1.00	
No change in frequency	0.69	0.47–1.01	0.80	0.54–1.17	0.81	0.55–1.18
More frequently	**0.72**	**0.53–0.98**	0.83	0.61–1.13	0.84	0.62–1.14

^1^Perceieved stress was measured using a using a modified version of the validated Perceived Stress Scale. The scale had four items. For more detail refer to the questionnaire section.

^2^Model set 1 is unadjusted. Model set 2 is adjusted for age, gender identity, and financial status change. Model set 3 is adjusted for age, gender identity, and financial status change, and other greenspace metrics: Models for greenspace space use and change in use are adjusted for proximity, and models for perceived greenspace proximity is adjusted for greenspace use.

^3^ Represents the participant’s perceived change at the time of survey response, relative to before the middle of March in year 2020.

Bolded RR and 95% CIs represent statistically significance (p-value <0.05).

### Associations between greenspace measures and changes in physical health

Table 6 shows RR (95% CI) estimates of associations between the greenspace metrics and worsened physical health. All RR estimates suggested a protective association with the greenspace measures. In fully-adjusted models, those who visited greenspaces most frequency (i.e., more than four times per week had 44% lower risk of reporting declines in physical health compared to those who visited greenspaces least frequently (fewer than 2 times per month, 95% CI: 0.34–0.93). However, there were not substantial differences in reported declines in physical health, comparing participants who visited greenspaces one to four times per week with those who visited greenspaces fewer than 2 times per month (RR: 1.06, 95% CI: 0.72–1.56). In fully adjusted models, compared to survey participants who reported reducing their frequency of greenspace use during the pandemic, those who reported visiting greenspaces with the same frequency had 41% lower risk of reporting declines in physical health (95% CI: 0.39–0.91). Those who increased their frequency of greenspace use were also protected against physical health declines; however, the protective association in this group was less substantial than for the participants who did not change their frequency of greenspace utilization (RR: 0.77, 95% CI: 0.56–1.06). RR estimates of association with greenspace proximity were close to null.

**Table 6:** Estimates of association between the greenspace measures and worsened physical health, comparing the survey period to before the start of pandemic-related social distancing measures in March of 2020^1^.

	Model Set 1		Model Set 2		Model Set 3	
	RR	95% CI	RR	95% CI	RR	95% CI
**Perceived proximity (Walking distance to nearest greenspace)**						
More than 10 minutes	1.00		1.00		1.00	
Less than 10 minutes	0.88	0.66–1.17	0.94	0.70–1.25	0.99	0.75–1.33
**Greenspace use (past 30 days)**						
Less than 2 times a month	1.00		1.00		1.00	
1–4 times a week	1.09	0.74–1.59	1.08	0.73–1.58	1.06	0.72–1.56
More than 4 times a week	**0.57**	**0.35–0.92**	**0.57**	**0.35–0.94**	**0.56**	**0.34–0.93**
**Greenspace use change** ^2^						
Less frequently	1.00		1.00		1.00	
No change in frequency	**0.54**	**0.40–0.84**	**0.58**	**0.38–0.90**	**0.59**	**0.39–0.91**
More frequently	0.75	0.55–1.03	0.77	0.56–1.1	0.77	0.56–1.06

^1^Model set 1 is unadjusted. Model set 2 is adjusted for age, gender identity, and financial status change. Model set 3 is adjusted for age, gender identity, and financial status change, and other greenspace metrics: Models for greenspace space use and change in use are adjusted for proximity, and models for perceived greenspace proximity is adjusted for greenspace use.

^2^Represents the participant’s perceived change at the time of survey response, relative to before the middle of March in year 2020.

Bolded RR and 95% CIs represent statistically significance (p-value <0.05).

### Sensitivity analyses

Results from the sensitivity analysis that used change in greenspace use compared to 12 months prior as the primary dependent variable were similar to those based on greenspace use changes compared to the period before the start of the pandemic (S2 Table). Results also remained relatively unchanged after additional adjustment for residence inside vs. outside Philadelphia (S3–S6 Tables).

## Discussion

This survey data from 485 adult residents living in one of the largest metropolitan areas of the United States during the COVID-19 pandemic contributes to evidence that greenspace may provide modest physical and mental health benefits. In particular, our analyses suggest that maintaining or increasing frequency of greenspace use was associated with modestly lower risk of experiencing decrements in physical and mental health, higher perceived stress, and greater feelings of loneliness during the pandemic.

Our results corroborate anecdotal reports of increased greenspace use during the pandemic [34–36] and are consistent with peer-reviewed research on this topic. Researchers in Australia found a 36% increase in survey respondents’ use of greenspace during public health restrictions compared to before [34]. Similarly, a study of Norwegian residents found a more than twofold increase in recreational use of outdoor spaces in the early stages of COVID-19 lockdown [35]. Similar results were reported elsewhere in Europe, including in Germany and the United Kingdom [36, 37]; however, these studies in European cities are not necessarily generalizable to the United States. A study in multiple cities in North Carolina had conflicting results, as the researchers found that urban park use declined during the pandemic with 56% of respondents reporting that they stopped or reduced park use [38].

The majority of respondents reported worsened mental health, greater feelings of loneliness, and higher perceived stress, but less than a third reported worsened physical health before versus mid-March. Previous literature has also identified increases in the prevalence of poor mental [25, 39–42] and physical health [43–45] during the pandemic. Moreover, a study conducted in Spain with 3041 participants found that COVID-19 pandemic restrictions were associated with decreased physical activity and declines in mental health [46].

Our results also contribute to a growing body of research investigating associations between greenspace exposure or use and physical and mental health outcomes during the COVID-19 pandemic. For example, consistent with our results, a study in South Korea found that participants who decreased greenspace use during the pandemic had 2.06 times the odds of mental health disorders compared to participants whose frequency of greenspace use decreased or did not change [12]. Jackson et al. found that frequent participation in outdoor play activities prior to the pandemic protected against decrements in subjective well-being during the pandemic in a survey distributed across the USA [47]. In a survey of 1188 adults living in Denver, Colorado, USA, Reid et al. found greenspace use was significantly associated with lower anxiety and depression [41]. Another study of 5218 people living in nine different countries situated in Europe, North, and South America, found that among participants under strict pandemic lockdown, those with access to viewing nature from a balcony reported better mental health and positive emotions compared to those who did not [48]. Moreover, in a study of participants with a mean age of 84 years old from the Lothian Birth Cohort, found a higher frequency of home gardening during the pandemic lockdown compared to pre-lockdown was associated with better self-rated physical health and mental health [49]. Likewise in a survey of 495 adults living in Germany, researchers found that those who owned gardens during the pandemic had substantially greater life satisfaction and mental well-being than non-garden owners [50].

In our study, surprisingly, we found that those who lived within closer walking distances of the nearest greenspace experienced higher risk of reporting decrements in worsened mental health and greater feelings of loneliness during the pandemic compared to those who lived further distances. It is possible that these results are explained by a suburban effect; a higher percentage of survey participants living in more suburban settings, outside the limits of Philadelphia, lived closer to the nearest greenspace. Living in these suburban areas may be associated with greater feelings of loneliness. For example, a survey of older adults living in the metropolitan Minneapolis region found that living further from the city is associated with enhanced feelings of social isolation and loneliness [51]. A potential mechanism for disparities in mental health according to urbanicity is described in a literature review by Gamm et al. The researchers highlight the potential barriers to mental health care that rural citizens face such as limited access to providers with sufficient expertise and training, as well as general stigma towards utilizing services or lack of awareness of mental health disorders [52]. Unfortunately, because of low statistical power, we were unable to fully explore these hypotheses by subsetting our analysis to Philadelphia city residents alone. We did, however, find that results remained unchanged even after adjusting for Philadelphia residence.

We acknowledge several limitations in this study. First, the study population is not generalizable to the entire population of the metropolitan Philadelphia region, or to other geographic areas. We used a convenience sampling approach to capture survey participants, advertising through personal or academic social networks and through various local community organizations, many of which focused on sustainability and environment, which could have led to selection bias and poor generalizability. Indeed, white, high-income, and highly educated women were over-represented in our participants. Even though the sociodemographic composition of the study population was homogeneous, the study area included a range of zip codes with varying levels of greenspace cover. Secondly, because our analyses were based on participant self-reports of most of the exposure variables, and the outcomes, there is the potential for recall bias, and possible exposure or outcome misclassification. Thus, we stress that these results reflect perceptions of mental and physical health outcomes, and of greenspace use or proximity. Because of the relatively small sample size and homogeneous study population, we did not have the statistical power to assess several factors, such as physical activity or race/ethnicity, as potential mediators or modifiers. Lastly, to keep the survey length reasonable, we did not collect data on other potential covariates, such as family circumstances, that could exacerbate a person’s negative response to the pandemic restrictions.

## Conclusion

This study contributes to the growing body of literature examining associations between greenspace use and physical and mental health outcomes during pandemic lockdowns. Our results suggest that maintaining greenspace use during the COVID-19 pandemic was associated with better mental and physical health outcomes. These results are important, as they imply that greenspace interventions should be designed in such a way to ensure that they are accessible and attractive. As well, this work implies the importance of ensuring that adults are given the time to use greenspaces, particularly during crisis situations, as a physical and mental health promotion tool.

## Supporting information

S1 File Statement of informed consent. (DOCX)

S2 File Survey on the impacts of greenspace utilization on health and well-being during a pandemic. (DOCX)

S1 Table Relationship between greenspace measures and health measures. * Statistically significant (p-value < 0.05).
(DOCX)

S2 Table Estimates of association between changes in greenspace use (during social distancing measures, compared to the prior year) and health outcomes. Estimates from model set 1 are unadjusted, estimates from model set 2 are adjusted for age, gender identity, and financial status change, and estimates from model set 3 are adjusted for age, gender identity, financial status change, and greenspace proximity. *Represents the participant’s perceived change at the time of survey response, relative to before the middle of March in year 2020. Bolded RR and 95% CIs represent statistically significance (p-value <0.05).
(DOCX)

S3 Table Estimates of association between the greenspace measures and mental health change, comparing the survey period to before the start of pandemic-related social distancing measures in March of 2020. Model set 1 is unadjusted. Model set 2 is adjusted for age, gender identity, and financial status change. Model set 3 is adjusted for age, gender identity, and financial status change, and other greenspace metrics: Models for greenspace space use and change in use are adjusted for proximity. Models for perceived greenspace proximity is adjusted for greenspace use. All models were adjusted for Philadelphia status. Bolded RR and 95% CIs represent statistically significance (p-value <0.05).
(DOCX)

S4 Table Estimates of association between the greenspace measures and physical health change, comparing the survey period to before the start of pandemic-related social distancing measures in March of 2020. Model set 1 is unadjusted. Model set 2 is adjusted for age, gender identity, and financial status change. Model set 3 is adjusted for age, gender identity, and financial status change, and other greenspace metrics: Models for greenspace space use and change in use are adjusted for proximity. Models for perceived greenspace proximity is adjusted for greenspace use. All models were adjusted for Philadelphia status. Bolded RR and 95% CIs represent statistically significance (p-value <0.05).
(DOCX)

S5 Table Estimates of association between the greenspace measures and loneliness change, comparing the survey period to before the start of pandemic-related social distancing measures in March of 2020. Model set 1 is unadjusted. Model set 2 is adjusted for age, gender identity, and financial status change. Model set 3 is adjusted for age, gender identity, and financial status change, and other greenspace metrics: Models for greenspace space use and change in use are adjusted for proximity. Models for perceived greenspace proximity is adjusted for greenspace use. All models were adjusted for Philadelphia status. Bolded RR and 95% CIs represent statistically significance (p-value <0.05).
(DOCX)

S6 Table Estimates of association between the greenspace measures and participant perceived stress. Model set 1 is unadjusted. Model set 2 is adjusted for age, gender identity, and financial status change. Model set 3 is adjusted for age, gender identity, and financial status change, and other greenspace metrics: Models for greenspace space use and change in use are adjusted for proximity, and models for perceived greenspace proximity is adjusted for greenspace use. All models were adjusted for Philadelphia status. Bolded RR and 95% CIs represent statistically significance (p-value <0.05).
(DOCX)
