# Supplementary material for: Associations of greenspace use and proximity with self-reported physical and mental health outcomes during the COVID-19 pandemic
Source: PLoS One. 2023 Mar 1;18(3):e0280837. doi: 10.1371/journal.pone.0280837 (PMC9977027; doi:10.1371/journal.pone.0280837)
Supplement: S1 File — (DOCX) [file pone.0280837.s001.docx]

**S1 File. Statement of informed consent**

Start of Block: Eligibility screening

Q58 We invite you to participate in a short survey that is being conducted researchers at the Drexel University Dornsife School of Public Health. Your responses will be used to study the health effects of using green spaces, such as parks and gardens, during the Covid-19 pandemic. The survey should take less than ten minutes for you to complete. 
Participating in the survey is completely voluntary. You are welcome to skip any questions that you prefer not to answer, and you can stop participating at any time. Choosing to not participate will not be held against you. Your survey responses will be completely anonymous, and we will not collect any identifying or contact information from you. We will not contact you again after you participate in the survey. There are no risks or benefits to participating in this survey. If you have any questions, please contact the Principal Investigator of this study, Dr. Leah Schinasi, MSPH, PhD at: lhs36@drexel.edu or (INSERT PHONE NUMBER).
This research is being overseen by an Institutional Review Board (“IRB”). An IRB is a group of people who perform independent review of research studies. You may talk to them at (267) 359-2471 or HRPP@drexel.edu if: - You have questions, concerns, or complaints that are not being answered by the research team. - You are not getting answers from the research team. - You cannot reach the research team. - You want to talk to someone else about the research.  - You have questions about your rights as a research subject.
By proceeding to the next page, you are consenting to participate in this study.
