# Supplementary material for: Associations of greenspace use and proximity with self-reported physical and mental health outcomes during the COVID-19 pandemic
Source: PLoS One. 2023 Mar 1;18(3):e0280837. doi: 10.1371/journal.pone.0280837 (PMC9977027; doi:10.1371/journal.pone.0280837)
Supplement: S2 File — (DOCX) [file pone.0280837.s002.docx]

**S2 File. Survey on the Impacts of greenspace utilization on health and well-being during a pandemic.**

Q61 Are you 18 years of age or older?

- Yes (1)
- No (2)

Skip To: End of Survey If Are you 18 years of age or older? = No

| 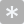 |
| --- |

Q74 What is today's date?

________________________________________________________________

End of Block: Eligibility screening

Start of Block: Greenspace Use & Access

Q65 In the next set of questions, we will ask you some questions about your use of green spaces or natural spaces. Green spaces or natural spaces are defined as areas containing trees, grass, shrubs, plants, rivers, lakes, ponds, and/or oceans.

Q42 Thinking back to the past 30 days, what type of green or natural space have you generally visited? (Please select all that apply)

- School playground (1)
- City public park (2)
- Hiking trail / forest (3)
- Biking / running trail (4)
- Community garden (5)
- Private property / personal lawn or garden (6)
- River, lake, or beach (7)
- Private garden (8)
- Private yard (10)
- I have not visited any type of green space (11)

Q40 Over the past 30 days, how often have you visited a green or natural space?

- More than 4 times per week (1)
- 2-4 times per week (2)
- 1-2 times per week (3)
- 1-2 times per month (4)
- Almost never (5)

Q39 Compared to before the start of the Coronavirus outbreak in mid-March, would you say that you visit green or natural spaces:

- More often (1)
- The same amount (2)
- Less often (3)

Q68 Compared to this time last year (12 months ago), would you say that you visit green or natural spaces:

- More often (1)
- The same amount (2)
- Less often (3)

Q67 Does your home or property have a garden, lawn, or tree? (select all that apply)

- Garden (1)
- Lawn (2)
- Tree (4)
- None of the above (6)

Q45 How long does it take you to walk to the green or natural space that is closest to your home?

- Fewer than 5 minutes (1)
- 5-9 minutes (2)
- 10-19 minutes (3)
- 20-30 minutes (4)
- more than 30 minutes (5)
- I cannot walk to a greenspace (6)
- I do not know of any nearby greenspaces (7)

Q72 Do you generally feel safe while visiting the green or natural spaces in your neighborhood:

|  | Yes (1) | No (2) |
| --- | --- | --- |
| During the day (1) |  |  |
| At night (2) |  |  |

Display This Question:

If Over the past 30 days, how often have you visited a green or natural space? = 1-2 times per month

Or Over the past 30 days, how often have you visited a green or natural space? = Almost never

Q48 What are the reasons that you are unable or reluctant to visit green spaces or natural spaces? (select all that apply)

- I don't have time (1)
- Green spaces are too far from my home (2)
- Lack of child play facilities (3)
- I am concerned about safety (poor lighting at night in parks, crime, homelessness) (4)
- I feel out of place or unwelcome (5)
- Too many dogs (6)
- Dogs not allowed (7)
- Poor quality parks, green spaces, or natural spaces (8)
- Too much litter / trash in the park, green space, or natural spaces (9)
- I'm worried about pollution or pesticides (10)
- The greenspace bothers my allergies. (11)
- I do not have access to transportation. (12)
- Worried about Coronavirus infection (13)
- I am not reluctant to visit green or natural spaces (14)

Q73 What types of activities do you engage in when you visit green spaces? (Select all that apply)

- Walking/hiking (1)
- Running (2)
- Bicycling (3)
- Team games and sports (4)
- Gardening (5)
- Using playgrounds/playing with children (6)
- Reading/relaxing (7)
- Other (8) ________________________________________________

End of Block: Greenspace Use & Access

Start of Block: Health Habits and Wellbeing

Q32 Compared to before the start of the coronavirus outbreak, would you say that you exercise:

- More often (1)
- Less often (2)
- I have not changed my exercise frequency (3)

Q35 How have the following changed since the start of the shutdown in mid-March, if at all?

|  | Improved (1) | No different (2) | Gotten worse (3) |
| --- | --- | --- | --- |
| Your physical health (1) |  |  |  |
| Your mental health (2) |  |  |  |
| Your financial well-being (3) |  |  |  |
| Feelings of loneliness (4) |  |  |  |

Q34 In the past 30 days, have you used tobacco or electronic cigarettes?

- Yes (1)
- No (2)

Q52 In the past 30 days, how often have you felt that you were unable to control the important things in your life?

- Never (1)
- Almost never (2)
- Sometimes (3)
- Fairly often (4)
- Very often (5)

Q53 In the past 30 days, how often have you felt confident about your ability to handle your personal problems?

- Never (1)
- Almost never (2)
- Sometimes (3)
- Fairly often (4)
- Very often (5)

Q54 In the past 30 days, how often have you felt that things were going your way?

- Never (1)
- Almost never (2)
- Sometimes (3)
- Fairly often (4)
- Very often (5)

Q55 In the past 30 days, how often have you felt difficulties were piling up so high that you could not overcome them?

- Never (1)
- Almost never (2)
- Sometimes (3)
- Fairly often (4)
- Very often (5)

Q64 In the past 30 days, how often have you felt lonely?

- Never (1)
- Almost never (2)
- Sometimes (3)
- Fairly often (4)
- Very often (5)

Q71 In the past 30 days, not including any work-related (paid) responsibilities, have you been responsible for:(select all that apply)

- Infant or child care (1)
- Homeschooling/Facilitating virtual education for grade school children (K-12) (2)
- Caring for adults (e.g., an elderly relative) (3)
- None of the above (4)

End of Block: Health Habits and Wellbeing

Start of Block: Occupation

Q26 In the past 30 days, what has been your occupational status? (select all that apply)

- Working outside the home, for pay (1)
- Working from home, for pay (2)
- Furloughed (3)
- Student (6)
- Unemployed (7)
- Retired (8)

Q69 In the past 30 days have you come into direct contact with members of the public, through your work?

- Yes (1)
- No (2)

Q40 Over the past 30 days, have you worked in any of the following industries? (please select the choice that applies to you)

- In person health or patient care (e.g., nurse, nursing assistant, physician, dentist, EMS etc.) (1)
- Public service (e.g., police officer, firefighter) (2)
- Restaurant industry (e.g., waitstaff) (3)
- Retail / Grocery (4)
- Public transit (5)
- In person education (k-12) or childcare (6)
- None of the above (7)

Display This Question:

If In the past 30 days, what has been your occupational status? (select all that apply) = Working outside the home, for pay

Q27 Do you wear a mask at all times while you are working?

- Yes (1)
- No (2)

End of Block: Occupation

Start of Block: Health History & Risks

Q31 How would you describe your general health, currently?

- Excellent (1)
- Very Good (2)
- Good (3)
- Fair (4)
- Poor (5)

Q32 Since March 2020, have you experienced any of the following? (select all that apply)

- New loss of taste or smell (1)
- New rash on finger or toes (2)
- New difficulty breathing (3)
- New cough (4)
- Unusual Fatigue (5)
- Fever about 100.0 F or feeling feverish (chills, body aches) (6)
- Vomiting or Diarrhea (7)
- Loss of appetite (8)
- None of the above (9)

Q49 Have you ever been tested for COVID-19?

- Yes (1)
- No (2)

Skip To: Q35 If Have you ever been tested for COVID-19? = No

Q33 Did you ever test positive for COVID-19?

- Yes (1)
- No (2)

Skip To: Q35 If Did you ever test positive for COVID-19? = No

Q34 Have you ever been hospitalized for COVID-19?

- Yes (1)
- No (2)

Q35 Has anyone else living in your household ever tested positive for COVID-19?

- Yes (1)
- No (2)

Display This Question:

If Did you ever test positive for COVID-19? != Yes

Q38 To your knowledge, have you ever had direct contact with someone who was infected with the COVID-19 virus (at the time that they were infected)?”

- Yes (1)
- No (2)

Display This Question:

If To your knowledge, have you ever had direct contact with someone who was infected with the COVID-... = Yes

Q70 Were you wearing a mask or face covering at the time that you came into contact with the person or people infected with the COVID-19 virus?

- Yes, all of the time (1)
- No, none of the time (2)
- Some of the time (4)

End of Block: Health History & Risks

Start of Block: Neighborhood environment

Q37 Please think about your neighborhood over the past 30 days.For each statement, please indicated “strongly agree,” “agree,” “disagree,” “strongly disagree,” or “don’t know.”

|  | Strongly agree (1) | Agree (2) | Disagree (3) | Strongly disagree (4) | Don't know (5) |
| --- | --- | --- | --- | --- | --- |
| My neighborhood is clean (1) |  |  |  |  |  |
| My neighborhood is safe (2) |  |  |  |  |  |
| The police presence in my neighborhood is more beneficial than stressful (3) |  |  |  |  |  |
| There is a lot of crime in my neighborhood (4) |  |  |  |  |  |
| There is too much drug use in my neighborhood (5) |  |  |  |  |  |
| There are lots of abandoned buildings or lots in my neighborhood (6) |  |  |  |  |  |

End of Block: Neighborhood environment

Start of Block: Sociodemographics

Q21 Do you have access to a vehicle, which you can use for transportation?

- Yes (1)
- No (2)
- Sometimes (3)

Q63 What is your current age?

- 18 - 24 (1)
- 25 - 34 (2)
- 35 - 44 (3)
- 45 - 54 (4)
- 55 - 64 (5)
- 65 - 74 (6)
- 75 - 84 (7)
- 85 or older (8)

| 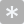 |
| --- |

Q17 How many bedrooms are in your home?

________________________________________________________________

| 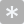 |
| --- |

Q18 How many people live in your home, including yourself?

________________________________________________________________

Q22 Do you or your family own or rent your home?

- Own (1)
- Rent (2)
- Neither (3)

Q24 What is your highest level of educational attainment?

- Less than high school graduate (1)
- High school graduate or GED (2)
- Some College, No Degree (3)
- Associate’s Degree (4)
- Bachelor’s Degree (5)
- Master’s Degree (6)
- Doctoral or Professional (MD, JD, PhD, etc.) Degree (7)

Q12 What is your race?

- American Indian or Alaska Native (1)
- Asian (2)
- Black or African-American (3)
- Native Hawaiian or Other Pacific Islander (4)
- White (5)
- Other: (6) ________________________________________________

Q13 Do you identify as Hispanic or Latinx?

- Yes (1)
- No (2)

Q66 What is your gender?

- Male (1)
- Female (2)
- Non-binary (3)
- Gender non-conforming (4)
- Other (5) ________________________________________________

Q25 What is your annual household income?

- $0-14,999 (1)
- $15,000-29,999 (2)
- $30,000-49,999 (3)
- $50,000-69,999 (4)
- $70,000-99,999 (5)
- $100,000-129,999 (6)
- $130,000-169,999 (7)
- $170,000-210,000 (8)
- greater than $210,000 (9)

End of Block: Sociodemographics
