# Supplementary material for: Associations of greenspace use and proximity with self-reported physical and mental health outcomes during the COVID-19 pandemic
Source: PLoS One. 2023 Mar 1;18(3):e0280837. doi: 10.1371/journal.pone.0280837 (PMC9977027; doi:10.1371/journal.pone.0280837)
Supplement: S1 Table — * Statistically significant (p-value < 0.05). (DOCX) [file pone.0280837.s003.docx]

| **S1 Table. Relationship between greenspace measures and health measures** | | | | |
| --- | --- | --- | --- | --- |
|  | **Worsened Mental Health** | **Higher Perceived Stress** | **Worsened Loneliness** | **Worsened Physical Health** |
| **Worsened Mental Health** | 1 | 0.36 * | 0.50 * | 0.27 * |
| **Higher Perceived Stress** | 0.36 * | 1 | 0.30 * | 0.22 * |
| **Worsened Loneliness** | 0.50 * | 0.29* | 1 | 0.14 |
| **Worsened Physical Health** | 0.27 * | 0.22* | 0.14 | 1 |

* Statistically significant (p-value < 0.05)
