# Supplementary material for: Associations of greenspace use and proximity with self-reported physical and mental health outcomes during the COVID-19 pandemic
Source: PLoS One. 2023 Mar 1;18(3):e0280837. doi: 10.1371/journal.pone.0280837 (PMC9977027; doi:10.1371/journal.pone.0280837)
Supplement: S2 Table — Estimates from model set 1 are unadjusted, estimates from model set 2 are adjusted for age, gender identity, and financial status change, and estimates from model set 3 are adjusted for age, gender identity, financial status change, and greenspace proximity. *Represents the participant’s perceived change at the time of survey response, relative to before the middle of March in year 2020. Bolded RR and 95% CIs represent statistically significance (p-value <0.05). (DOCX) [file pone.0280837.s004.docx]

| **S2 Table. Estimates of association between changes in greenspace use (during social distancing measures, compared to the prior year) and health outcomes.** Estimates from model set 1 are unadjusted, estimates from model set 2 are adjusted for age, gender identity, and financial status change, and estimates from model set 3 are adjusted for age, gender identity, financial status change, and greenspace proximity. *Represents the participant’s perceived change at the time of survey response, relative to before the middle of March in year 2020. Bolded RR and 95% CIs represent statistically significance (p-value <0.05). | | | | | | |
| --- | --- | --- | --- | --- | --- | --- |
|  | **Model Set 1** | | **Model Set 2** | | **Model Set 3** | |
| **Worsened Mental Health*** | RR | 95% CI | RR | 95% CI | RR | 95% CI |
| Less frequently | 1.00 |  | 1.00 |  | 1.00 |  |
| No change in frequency | **0.65** | **0.51- 0.83** | **0.71** | **0.56- 0.91** | **0.72** | **0.56-0.92** |
| More frequently | **0.76** | **0.64- 0.90** | **0.78** | **0.66- 0.93** | **0.78** | **0.65-0.92** |
| **Worsened Physical Health*** | RR | 95% CI | RR | 95% CI | RR | 95% CI |
| Less frequently | 1.00 |  | 1.00 |  | 1.00 |  |
| No change in frequency | **0.57** | **0.38-0.88** | **0.58** | **0.38-0.88** | **0.57** | **0.37-0.88** |
| More frequently | **0.72** | **0.53-0.97** | **0.69** | **0.51-0.94** | **0.69** | **0.51-0.95** |
| **Worsened Loneliness*** | RR | 95% CI | RR | 95% CI | RR | 95% CI |
| Less frequently | 1.00 |  | 1.00 |  | 1.00 |  |
| No change in frequency | **0.69** | **0.53-0.90** | **0.73** | **0.56-0.96** | **0.72** | **0.56-0.94** |
| More frequently | 0.87 | 0.71-1.05 | 0.90 | 0.75-1.08 | 0.91 | 0.75-1.09 |
|  |  |  |  |  |  |  |
| **Perceived Stress** | RR | 95% CI | RR | 95% CI | RR | 95% CI |
| Less frequently | 1.00 |  | 1.00 |  | 1.00 |  |
| No change in frequency | 0.79 | 0.55-1.15 | 0.90 | 0.62-1.31 | 0.90 | 0.62-1.31 |
| More frequently | **0.73** | **0.54- 0.99** | 0.78 | 0.58-1.06 | 0.80 | 0.60-1.08 |
