# Supplementary material for: Associations of greenspace use and proximity with self-reported physical and mental health outcomes during the COVID-19 pandemic
Source: PLoS One. 2023 Mar 1;18(3):e0280837. doi: 10.1371/journal.pone.0280837 (PMC9977027; doi:10.1371/journal.pone.0280837)
Supplement: S3 Table — Model set 1 is unadjusted. Model set 2 is adjusted for age, gender identity, and financial status change. Model set 3 is adjusted for age, gender identity, and financial status change, and other greenspace metrics: Models for greenspace space use and change in use are adjusted for proximity. Models for perceived greenspace proximity is adjusted for greenspace use. All models were adjusted for Philadelphia status. Bolded RR and 95% CIs represent statistically significance (p-value <0.05). (DOCX) [file pone.0280837.s005.docx]

| **S3 Table.** **Estimates of association between the greenspace measures and mental health change, comparing the survey period to before the start of pandemic-related social distancing measures in March of 2020.** Model set 1 is unadjusted. Model set 2 is adjusted for age, gender identity, and financial status change. Model set 3 is adjusted for age, gender identity, and financial status change, and other greenspace metrics: Models for greenspace space use and change in use are adjusted for proximity. Models for perceived greenspace proximity is adjusted for greenspace use. All models were adjusted for Philadelphia status. Bolded RR and 95% CIs represent statistically significance (p-value <0.05). | | | | | | |
| --- | --- | --- | --- | --- | --- | --- |
|  | **Model Set 1** | | **Model Set 2** | | **Model Set 3** | |
| **Perceived proximity (Walking distance to nearest greenspace)** | RR | 95% CI | RR | 95% CI | RR | 95% CI |
| More than 10 minutes | 1.00 |  | 1.00 |  | 1.00 |  |
| Less than 10 minutes | 0.97 | 0.82-1.14 | 1.00 | 0.86-1.18 | 0.99 | 0.84-1.18 |
|  |  |  |  |  |  |  |
| **Greenspace utilization frequency (past 30 days)** | RR | 95% CI | RR | 95% CI | RR | 95% CI |
| Less than 2 times a month | 1.00 |  | 1.00 |  | 1.00 |  |
| 1-4 times a week | 1.30 | 0.99-1.70 | 1.36 | 1.02-1.80 | 1.38 | 1.04-1.85 |
| More than 4 times a week | 1.19 | 0.89-1.60 | 1.26 | 0.93-1.71 | 1.28 | 0.93-1.75 |
|  |  |  |  |  |  |  |
| **Greenspace utilization frequency change (compared to before mid-March 2020) ^*^** | RR | 95% CI | RR | 95% CI | RR | 95% CI |
| Less frequently | 1.00 |  | 1.00 |  | 1.00 |  |
| No change in frequency | **0.67** | **0.53-0.85** | **0.75** | **0.59-0.95** | **0.74** | **0.58-0.95** |
| More frequently | **0.78** | **0.66-0.93** | 0.84 | 0.70-1.00 | 0.84 | 0.70-1.00 |
