# Supplementary material for: Associations of greenspace use and proximity with self-reported physical and mental health outcomes during the COVID-19 pandemic
Source: PLoS One. 2023 Mar 1;18(3):e0280837. doi: 10.1371/journal.pone.0280837 (PMC9977027; doi:10.1371/journal.pone.0280837)
Supplement: S6 Table — Model set 1 is unadjusted. Model set 2 is adjusted for age, gender identity, and financial status change. Model set 3 is adjusted for age, gender identity, and financial status change, and other greenspace metrics: Models for greenspace space use and change in use are adjusted for proximity, and models for perceived greenspace proximity is adjusted for greenspace use. All models were adjusted for Philadelphia status. Bolded RR and 95% CIs represent statistically significance (p-value <0.05). (DOCX) [file pone.0280837.s008.docx]

| **S6 Table. Estimates of association between the greenspace measures and participant perceived stress.** Model set 1 is unadjusted. Model set 2 is adjusted for age, gender identity, and financial status change. Model set 3 is adjusted for age, gender identity, and financial status change, and other greenspace metrics: Models for greenspace space use and change in use are adjusted for proximity, and models for perceived greenspace proximity is adjusted for greenspace use. All models were adjusted for Philadelphia status. Bolded RR and 95% CIs represent statistically significance (p-value <0.05) | | | | | | |
| --- | --- | --- | --- | --- | --- | --- |
|  | **Model Set 1** | | **Model Set 2** | | **Model Set 3** | |
|  | RR | 95% CI | RR | 95% CI | RR | 95% CI |
| **Perceived proximity (Walking distance to nearest greenspace)** |  |  |  |  |  |  |
| More than 10 minutes | 1.00 |  | 1.00 |  | 1.00 |  |
| Less than 10 minutes | 0.76 | 0.58-0.1.00 | 0.80 | 0.61-1.05 | 0.81 | 0.61-1.05 |
| **Greenspace use (past 30 days)** |  |  |  |  |  |  |
| Less than 2 times a month | 1.00 |  | 1.00 |  | 1.00 |  |
| 1-4 times a week | 0.88 | 0.62-1.24 | 0.90 | 0.64-1.27 | 0.88 | 0.62-1.23 |
| More than 4 times a week | 0.68 | 0.45-1.02 | 0.75 | 0.49-1.13 | 0.74 | 0.49-1.11 |
| **Greenspace use change^2^** |  |  |  |  |  |  |
| Less frequently | 1.00 |  | 1.00 |  | 1.00 |  |
| No change in frequency | 0.69 | 0.47-1.00 | 0.80 | 0.54-1.18 | 0.81 | 0.55-1.19 |
| More frequently | **0.72** | **0.53-0.97** | 0.84 | 0.62-1.14 | 0.85 | 0.63-1.15 |
